# The first case report of osteochondroma in a tetraplegic patient after spinal injury

**DOI:** 10.1097/MD.0000000000043309

**Published:** 2025-07-11

**Authors:** Saoussen Layouni, Sarra Ksibi, Ines Loubiri, Sinène Elfrigui, Sonia Jemni

**Affiliations:** a Faculty of Medicine of Sousse, University of Sousse, Sousse, Tunisia; b Department of Physical Medicine and Rehabilitation, Sahloul University Hospital, Sousse, Tunisia; c Faculty of Medicine of Sousse, Department of Family Medicine, University of Sousse, Sousse, Tunisia.

**Keywords:** benign tumor, case report, hip osteochondroma, recurrence, surgical treatment

## Abstract

**Rationale:**

Osteochondroma (OC) is a benign bone tumor commonly found near the ends of long bones. While spinal and hip localizations are rare, bilateral hip involvement is often associated with hereditary multiple exostoses, occurring in 30% to 90% of such cases. This report describes a unique case of bilateral hip OC developing after a spinal cord injury, in the absence of family history or hereditary multiple exostoses.

**Patient concerns:**

A 35-year-old male presented with painful bilateral hip swelling and restricted mobility following a rural accident that caused a C6–C7 vertebral fracture with bilateral anterior dislocation of the articular facets. He also exhibited spastic tetraplegia and flexion contractures, with signs suggestive of femoral nerve compression.

**Diagnoses:**

Initial imaging revealed myositis ossificans involving the iliopsoas and spinal muscles. A bone scan identified an immature osteoma. Histopathological examination following surgical excision confirmed the diagnosis of OC.

**Interventions:**

The patient underwent spinal surgery 2 days after the trauma, followed by admission to a physical medicine rehabilitation unit. Surgical resection of the left hip lesion improved flexion, and a similar procedure on the right hip yielded temporary improvement but led to complications, including infection requiring antibiotics and drainage.

**Outcomes:**

Despite surgical interventions, tumor recurrence occurred within 3 months. Imaging confirmed recurrent myositis ossificans and fluid collections. One year postinjury, neurological deterioration was noted, with spastic tetraplegia progressing from the C5 to C7 level and worsening lower limb spasticity. Valium was introduced for spasm management.

**Lessons:**

This case highlights a rare occurrence of bilateral hip OC following spinal cord injury and raises important questions about trauma-induced mechanisms in OC pathogenesis. It suggests the need for further investigation into the relationship between spinal trauma, heterotopic ossification, and tumor development.

## 1. Introduction

Osteochondroma (OC) is a cartilage-capped bony projection that develops on the external surface of the bone with a marrow cavity continuous with the underlying bone.^[1]^ It typically forms during the first decade of life and stops growing once skeletal maturity is reached.^[2]^ This benign bone tumor is usually found near the end of the long bones; in rare cases, it is found in the spine and hips. The bilateral hip form is often associated with hereditary multiple exostoses, with reports indicating its occurrence in 30% to 90% of patients with this condition.^[3]^

OC can present as a palpable mass that may cause pain owing to factors such as fractures through a pedunculated stalk, inflammation of the overlying bursa, impingement on surrounding structures, limited range of motion, and even hip joint subluxation. These tumors can lead to various complications that necessitate surgical intervention in some cases.^[4,5]^

Complete removal of the lesion with the cartilaginous cap is crucial to prevent recurrence and ensure optimal outcomes for patients with hip OCs.^[5,6]^

The exact origin of OCs remains controversial. Keith in 1920 attributed the formation of this bone tumor to a defect in the periosteal ring, which allowed for abnormal expansion and development of the lesion.^[7]^

There have been multiple case reports documenting the development of OCs after radiation.^[8,9]^

Several authors have linked peripheral trauma to the formation of an OC.^[10,11]^

In this case report, we presented the first case of a post-spinal cord injury bilateral hip OC of huge size in a 35-year-old male patient without a family history of OC or hereditary multiple exostosis, who required surgical treatment.

## 2. Case history

**Figure 1.**
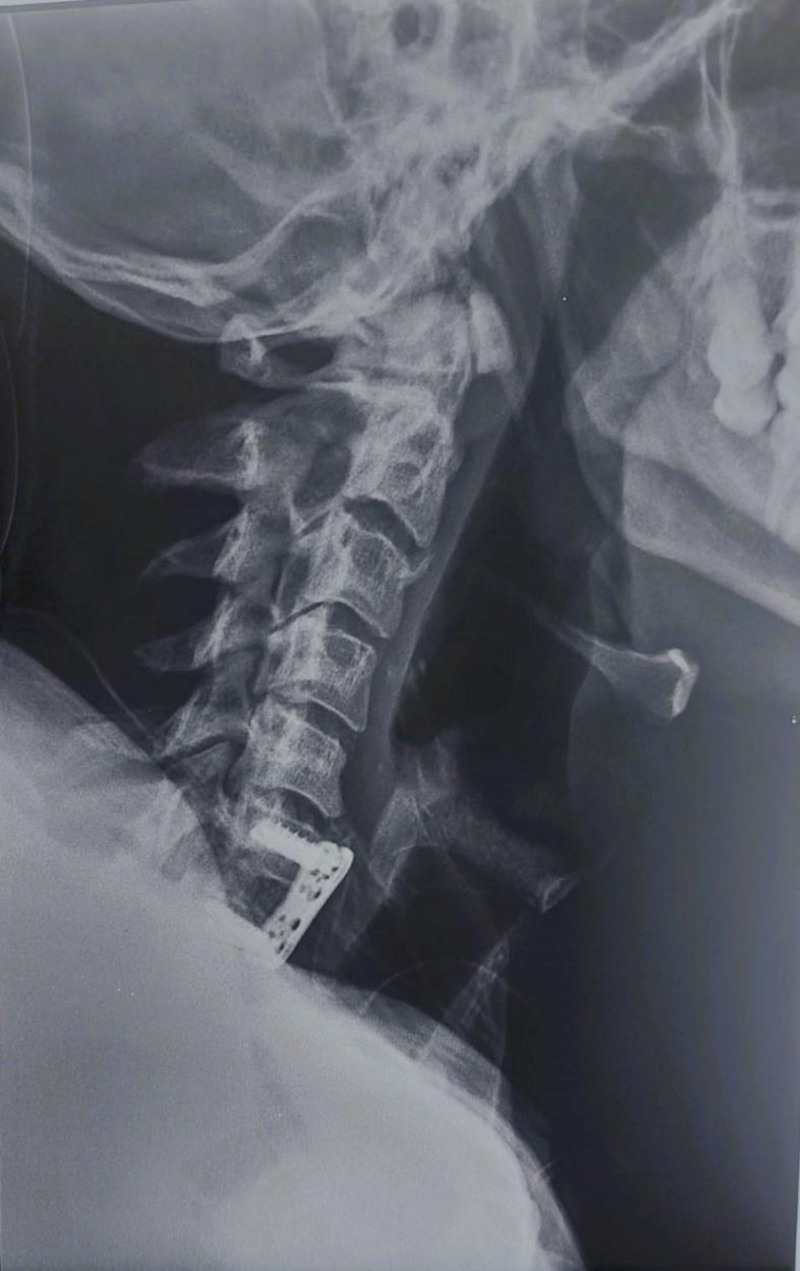
Lateral view of the cervical spine after surgery.

A 35-year-old patient who sustained a rural accident resulting in a vertebro-medullary trauma, specifically a C6-C7 fracture with anterolisthesis >80% and bilateral anterior dislocation of the articular facets, with a posterior wall displacement of approximately 80% at the C6/C7 level. He underwent surgery on day 2 post-trauma (Fig. 1). He was admitted to our physical medicine department 6 months after the accident. At the initial examination, he presented with spastic tetraplegia classified as AIS B at the C5 level; he has painful swelling with limitation of mobility of both hips, with 50° flexion contracture on the left and 30° on the right, blocked at 10° on the left and 20° on the right, with signs of femoral nerve compression as evidenced by the abolition of the patellar reflex bilaterally.

**Figure 2.**
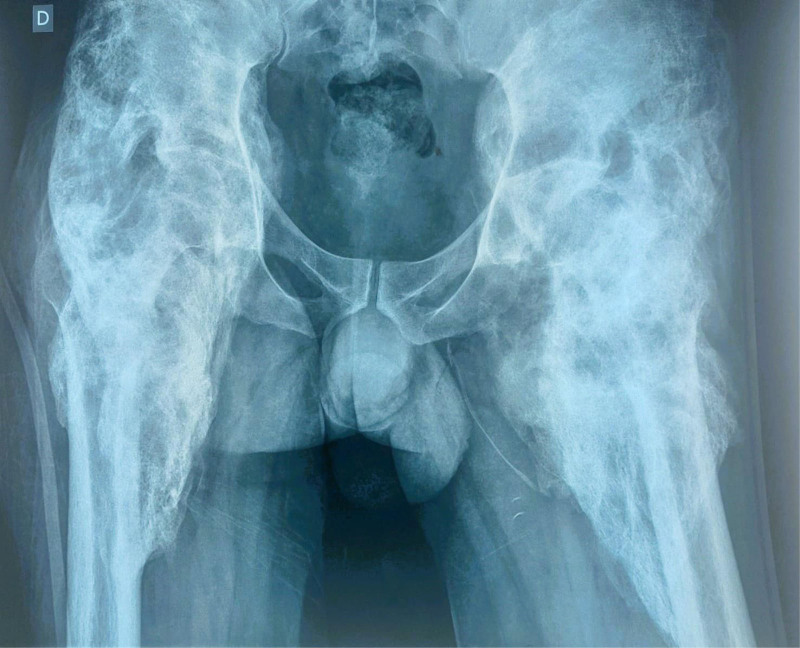
X-ray of the pelvis showing osteochondroma in both hips

**Figure 3.**
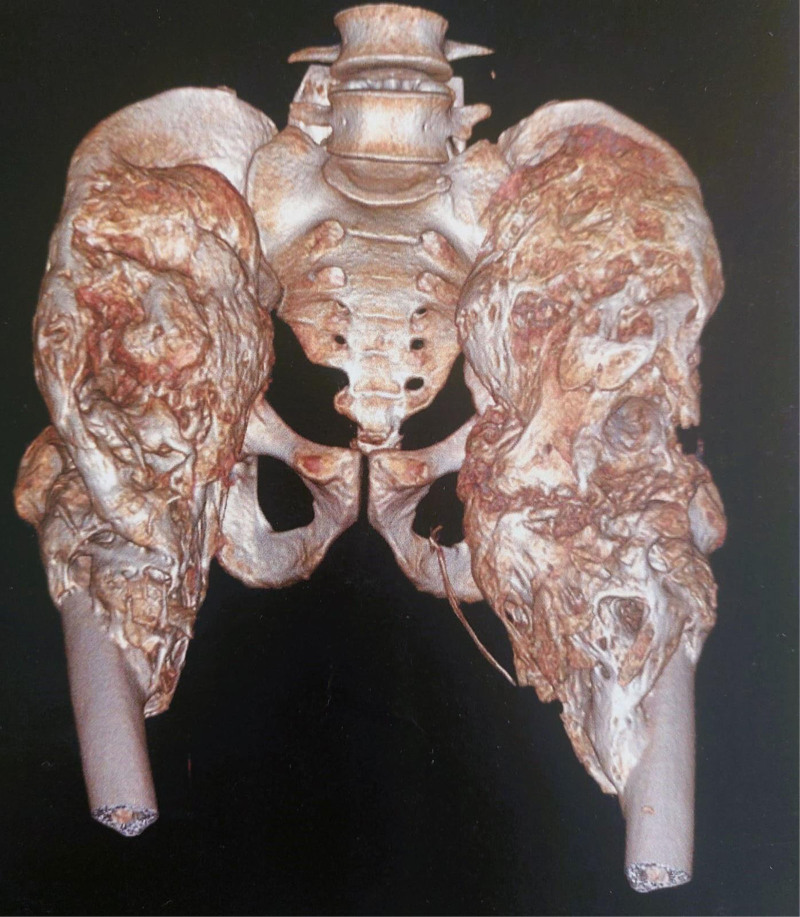
CT 3-dimensional reconstruction of the pelvis showing osteochondroma in both hips. CT = computed tomography.

**Figure 4.**
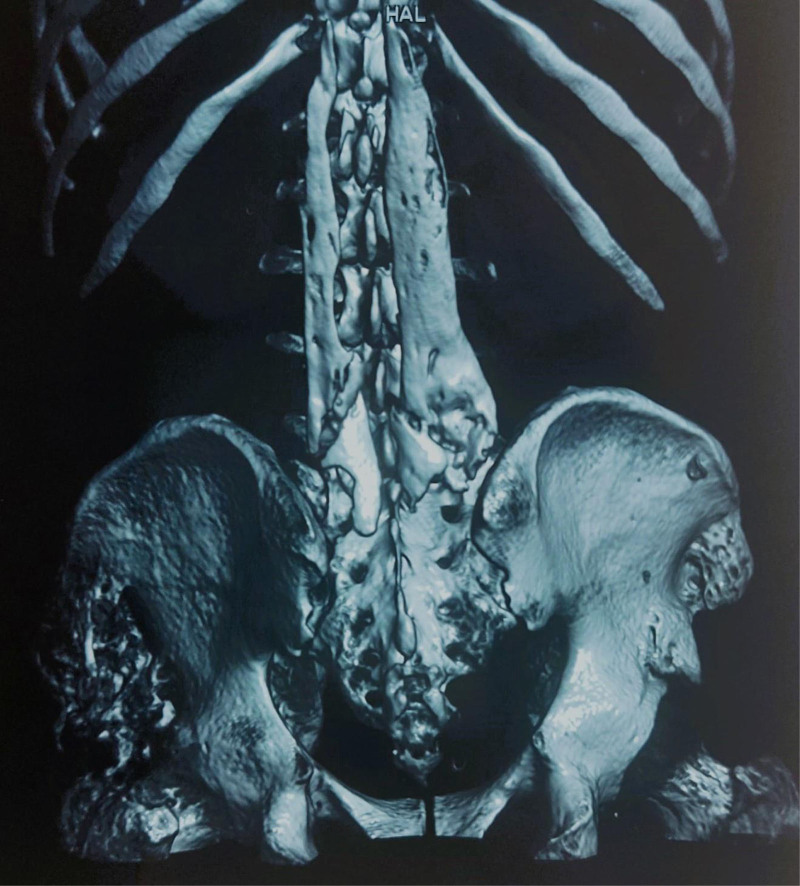
CT 3-dimensional reconstruction of spinal muscles and hips. CT = computed tomography.

**Figure 5.**
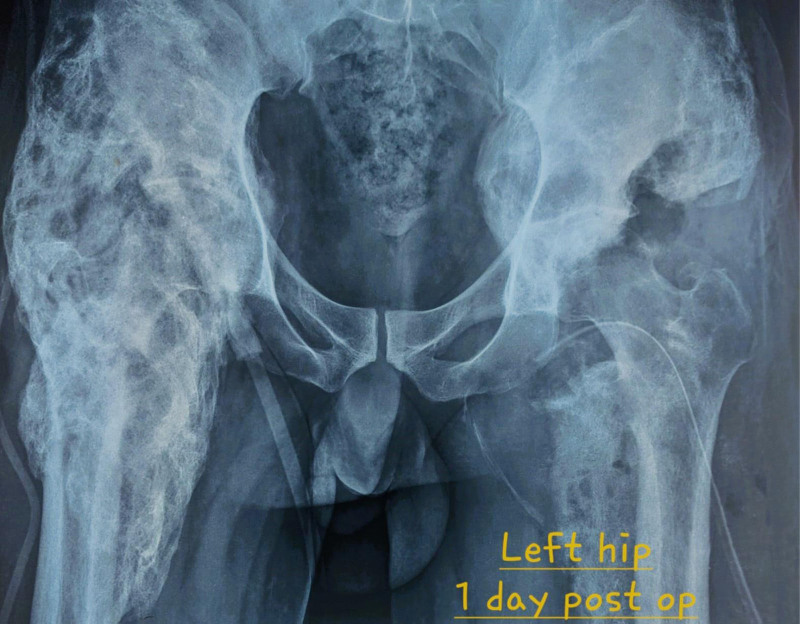
X-ray of the pelvis after surgery to remove the osteochondroma in the left hip (1 day post-op).

**Figure 6.**
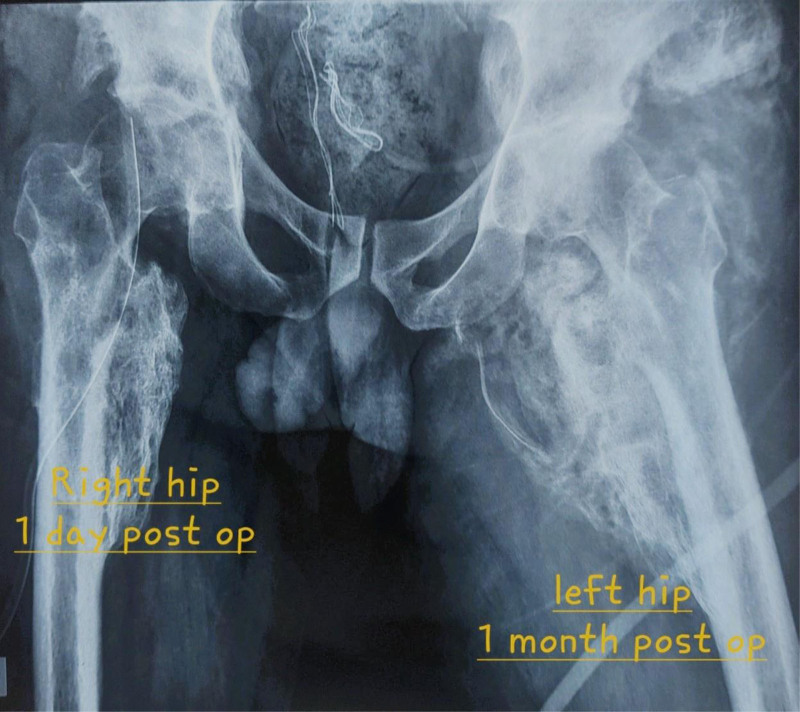
X-ray of the pelvis after the removal surgery of the osteochondroma in both hips.

A computed tomography (CT) scan of the pelvis revealed myositis ossificans with significant ossification of the iliopsoas and spinal muscle. After consulting with orthopedic specialists, a bone scan was requested, showing an immature osteoma (Figs. 2–4). He underwent resection of the osteoma in the left hip, which proceeded without complications, achieving 80° flexion (Fig. 5). The biopsy results for the osteoma showed the histological appearance of an OC. On postoperative day 26, an induration was observed at the surgical site, and an ultrasound revealed an 11-mm collection at the level of the greater trochanter. One month later, he underwent resection of the osteoma in the right hip (Fig. 6), achieving 100° flexion, which is also complicated by a collection at the level of the greater trochanter. He was started on antibiotics: Tienam, amikacin, and vancomycin for 14 days, followed by drainage of the collections.

In addition, the patient experienced multiple episodes of hypotension, managed initially with fluid resuscitation and later, upon the intensivists’ recommendation, with norepinephrine. Cardiological examination revealed no abnormalities. Given the recurrent episodes, an endocrinological consultation was sought, and the patient was switched to hydrocortisone (Cortef) following cortisol level assessment.

Moreover, the patient presented with respiratory distress. A CT angiography revealed a lobar and segmental pulmonary embolism in the left lower lobe without signs of pulmonary hypertension, accompanied by a pleural effusion and a moderately abundant anterior left pneumothorax, for which there was no indication for thoracentesis.

Furthermore, due to the persistence of orthostatic hypotension episodes, Cortef was discontinued, and Heptamyl was prescribed to treat the hypotension due to central dysautonomia. Despite the resection of the osteomas, there was a reappearance of formations on both sides 3 months later. A pelvic CT scan showed the aspect of myositis ossificans in the vertebral muscles and both hips with a larger collection near these areas: on the left measuring 89 × 55 mm and 86 × 37 mm on the right. Orthopedically, bilateral hip flexion was at 80°, blocked in Flexum at 25° on the right and 15° on the left, with the possibility of sitting at 90° in a wheelchair. After 3 months, both hips were fixed at 25° in flexion and had an abduction range of 0°, and the ability to sit was lost.

**Figure 7.**
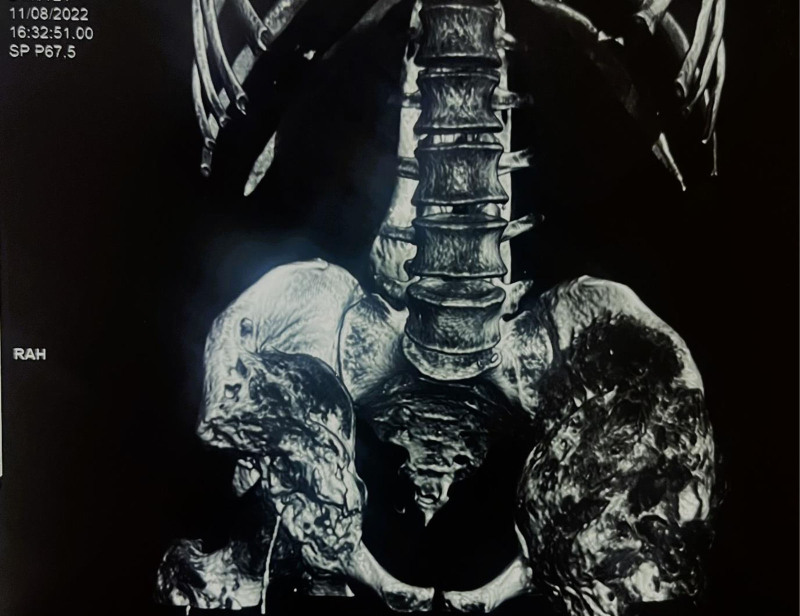
CT 3-dimensional reconstruction of spinal muscles and hips, a year after surgical treatment, showing reoccurrence of the tumor. CT = computed tomography, RAH = Rehaussement annulaire en Halo.

After a year from the accident (Fig. 7), the tetraplegia evolved from AISB (ASIA Impairment Scale [AIS]; B, Sensory Incomplete) at the C5 level to the C7 level, with generalized spasticity in the lower limbs bilaterally, which is functionally troublesome. The patient was placed on Valium to relieve spasms, with a Penn Spasticity Score of 2.

## 3. Discussion

OC constitutes the predominant form of benign osseous neoplasm originating from cartilaginous tissue. It comprises approximately 36% of all benign bone neoplasms and 8.5% of the total spectrum of bone tumors.^[12]^ Research conducted in Iran indicated that OC is the most prevalent variant of bone tumor, representing 35% of cases within the nation.^[13]^ These neoplasms typically manifest during the initial 2 decades of an individual’s life.

OCs predominantly occur at the metaphysis, in proximity to the growth plate of a long bone, and possess the capacity to develop within any bone that is formed via endochondral ossification.^[14]^ The lesion presents as an external bony protrusion upon the surface of the bone, composed of normal spongiosa and cortex, which continues seamlessly with the underlying healthy bone structure. This bony outgrowth is enveloped by hyaline cartilage, which is significantly thicker in the population of growing children and adolescents. As the process of skeletal maturation advances, the lesion frequently attains a state of stabilization, and the cartilaginous cap may undergo complete regression.^[15]^ The extent of calcification present within the cartilaginous cap demonstrates significant variability.

OCs can present as solitary or multiple lesions.^[14]^ Solitary OCs are sporadic and occur without a genetic predisposition. On the other hand, an autosomal dominant disorder is usually responsible for multiple OCs affecting different family members. Regardless of whether they are solitary or multiple, these lesions exhibit identical radiographic and pathological characteristics.

The etiology of solitary OCs remains enigmatic. While certain clinical presentations and the infrequent incidence of malignant transformation imply a neoplastic mechanism, alternative hypotheses suggest a congenital developmental origin.^[14]^ Numerous theorists contend that OCs develop from ectopic physeal cartilage that is displaced during the process of bone growth. This displaced cartilage subsequently undergoes endochondral ossification, thereby emulating the normal bone growth process.^[14]^ It is thought that a fragment of the physis pushes through the periosteal bone cuff surrounding the growth plate (Ranvier encoche), leading to growth on the bone’s surface.^[14,15]^

Another theory says that an OC arises from cartilaginous metaplasia within the periosteal membrane. Recent studies have elucidated that mutations in the exostosin glycosyltransferase 1 gene serve as a pivotal factor in the etiology of solitary OCs,^[16]^ resulting in abnormal cartilage proliferation and disruptions in signaling cascades such as bone morphogenetic protein and fibroblast growth factor pathways. Added to that, microRNAs have a role in the development of the tumor, opening the way to a potential treatment through miRNA regulation. However, in certain cases, OC may appear after a trauma or irradiation.^[17,18]^

In a comparable case involving a 35-year-old male subject who sustained a spinal injury, he exhibited clinical manifestations including difficulty in maintaining an upright seated posture and restrictions in hip joint mobility, which were attributable to the presence of bilateral OCs in the hip region, emerging within 6 months post-trauma.

OCs situated in proximity to the knee and hip joints frequently present without symptoms. Nevertheless, they possess the potential to exert pressure on neighboring vascular structures or nerves, resulting in a spectrum of clinical presentations.^[19]^ Such complications may encompass femoral anteversion, limitations in hip flexion, acetabular dysplasia leading to subsequent hip subluxation, knee valgus deformity, and various angular limb deformities.^[20]^

While this case provides valuable insights into the presentation and management of bilateral hip OCs following spinal cord injury, it has several strengths and limitations. As the first reported case of bilateral hip OCs of significant size in a patient with spinal cord injury, it offers novel insights into a rare clinical scenario. The study provides detailed clinical, radiological, and histopathological findings, supported by a multidisciplinary approach involving orthopedics, endocrinology, and intensive care, which emphasizes comprehensive care for complex cases. In addition, the documentation of surgical challenges, functional outcomes, and complications offers practical guidance for managing similar conditions. However, as a single case report, the findings lack generalizability and long-term follow-up, limiting insights into recurrence rates and functional outcomes over time. The unclear etiology of the OCs, compounded by the absence of molecular or genetic analysis, hinders a definitive understanding of their development post-trauma. Moreover, the recurrence of lesions and postoperative complications raises questions about surgical and perioperative management, which are not fully addressed. Imaging findings, therapeutic challenges, and the impact of spasticity and hypotension are discussed but not deeply analyzed. Last, the absence of comparisons with other similar cases or controls limits the broader applicability of the conclusions. Future research involving larger cohorts and long-term follow-up is needed to better understand this rare condition and improve patient outcomes.

## 4. Conclusion

To conclude, the presented case demonstrates a bilateral OC of the hip that required surgical treatment. However, within 3 months, the tumor reappeared in an even larger size. OC pathogenesis involves a complex interplay of genetic susceptibility, disruptions in bone development, and the potential for malignant transformation, particularly in cases of hereditary multiple OCs.

What could be the cause of an OC following a spinal cord injury? Are there specific stages or alterations in bone that contribute to its development?

## Author contributions

**Conceptualization:** Saoussen Layouni, Sarra Ksibi, Ines Loubiri, Sinène Elfrigui, Sonia Jemni.

**Methodology:** Saoussen Layouni, Sarra Ksibi.

**Project administration:** Saoussen Layouni, Sarra Ksibi, Ines Loubiri, Sinène Elfrigui, Sonia Jemni.

**Supervision:** Saoussen Layouni, Ines Loubiri, Sinène Elfrigui, Sonia Jemni.

**Validation:** Saoussen Layouni, Ines Loubiri, Sinène Elfrigui, Sonia Jemni.

**Visualization:** Saoussen Layouni.

**Writing—review & editing:** Saoussen Layouni, Sarra Ksibi, Ines Loubiri, Sinène Elfrigui, Sonia Jemni.

**Data curation:** Saoussen Layouni, Sarra Ksibi.

**Investigation:** Saoussen Layouni, Sarra Ksibi.

**Resources:** Saoussen Layouni, Sarra Ksibi.

**Software:** Sarra Ksibi.

**Writing—original draft:** Saoussen Layouni, Sarra Ksibi.
